# Supplementary material for: Analysis of the 5′ Untranslated Region Length-Dependent Control of Gene Expression in Maize: A Case Study with the ZmLAZ1 Gene Family
Source: Genes (Basel). 2024 Jul 29;15(8):994. doi: 10.3390/genes15080994 (PMC11353600; doi:10.3390/genes15080994)
Supplement: Supplementary file 1 [file genes-15-00994-s001.zip › Supplemental Data0702.pdf]

Supplemental Figures

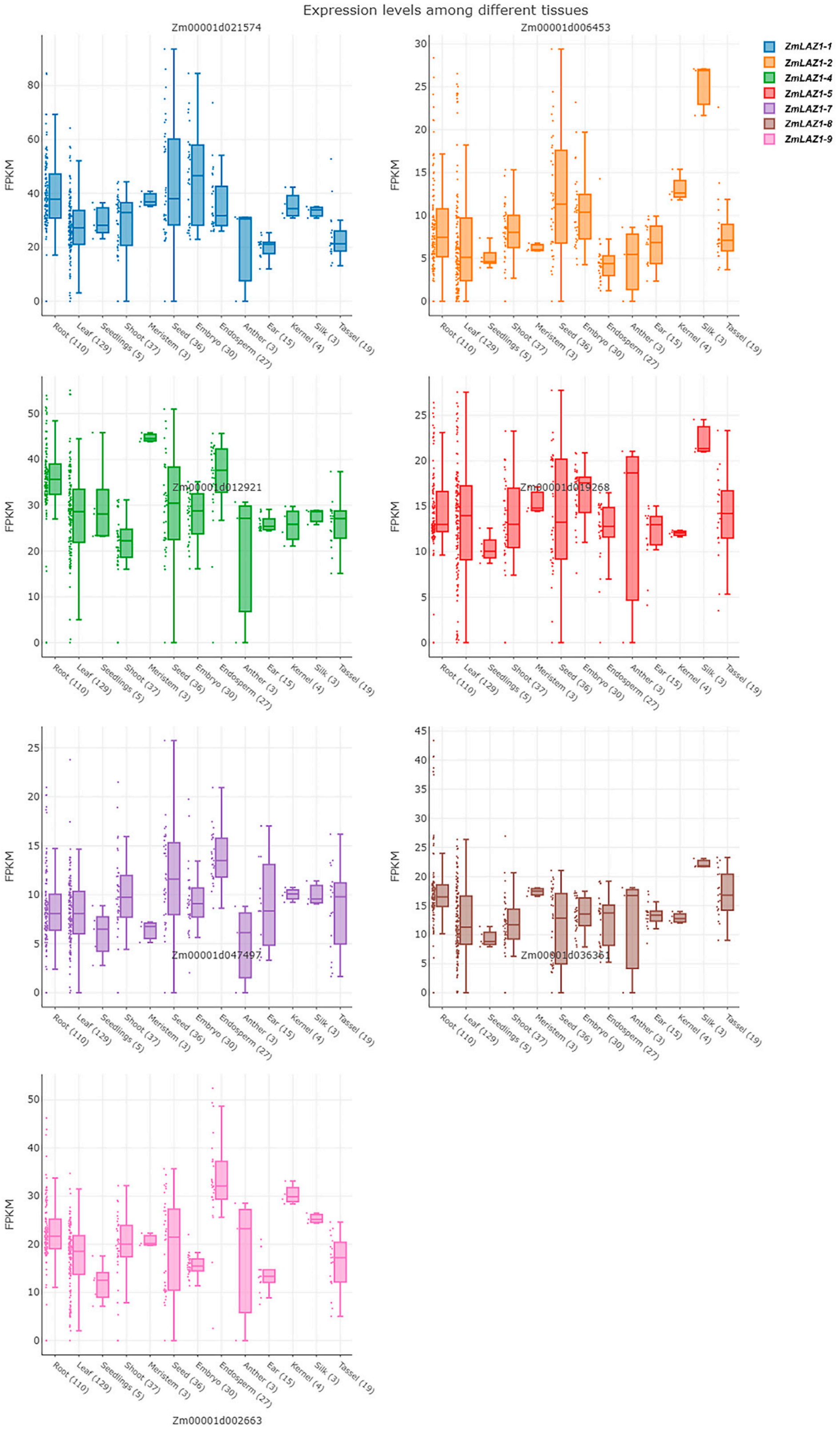

Supplemental Figure S1. Expression abundance of the *ZmLAZ1* family members in different maize tissues in the PPRD database.

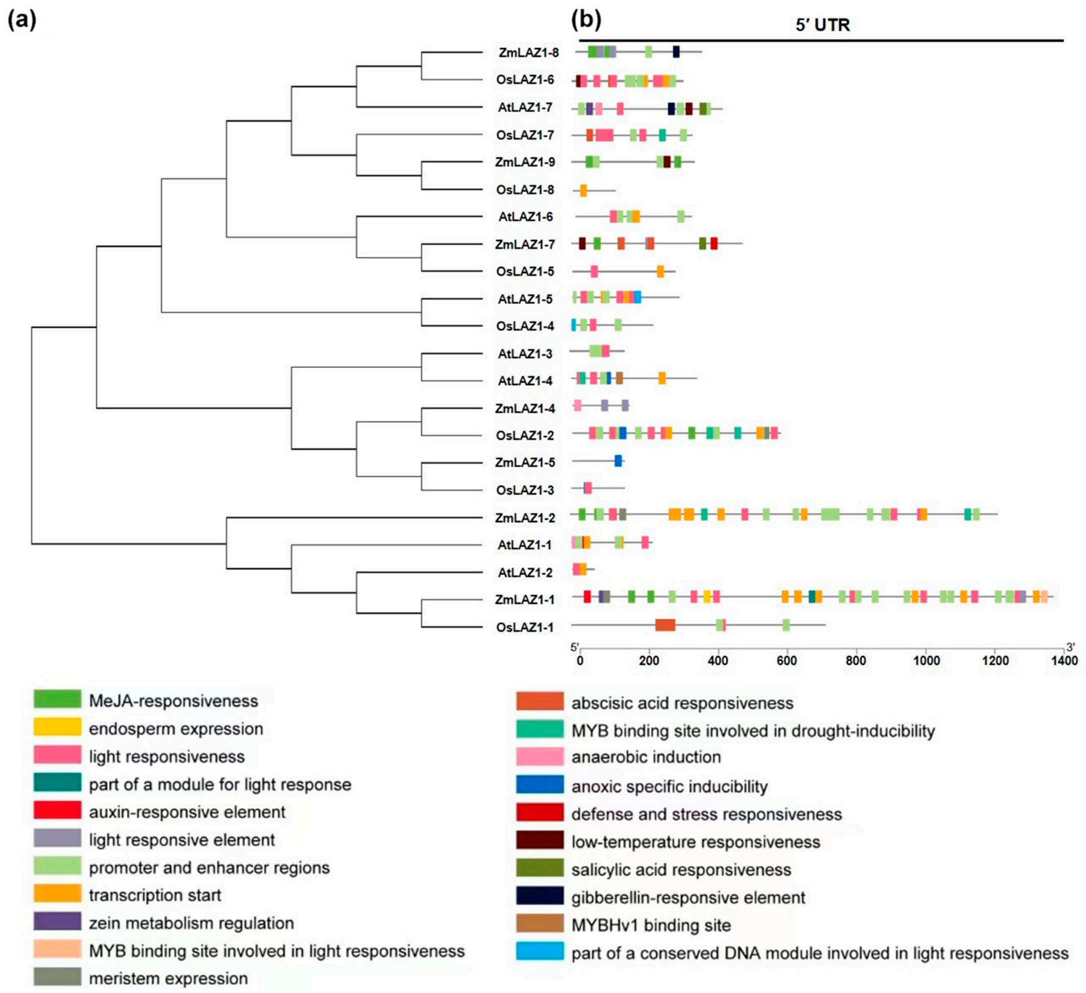

Supplemental Figure S2. Phylogenetic analysis of the 5′ UTR-containing *LAZ1* family in maize, rice, and arabidopsis and their 5′ UTR *cis*-elements. (**a**) Phylogenetic analysis of the *LAZ1* family. (**b**) The *cis*-acting elements of the 5′ UTR region, and different color blocks represent different elements.
